# Monoclonal Antibody Treatment for Pediatric Asthma: Current Evidence and Findings From a Delaware Cohort Study

**DOI:** 10.7759/cureus.97780

**Published:** 2025-11-25

**Authors:** Samantha Y Starkey, Abigail Strang, Elizabeth Brown, Alana B Jones, Vandna Passi, Aaron Chidekel

**Affiliations:** 1 Pediatrics, Nemours Children's Hospital, Delaware, Wilmington, USA; 2 Pulmonary Medicine, Nemours Children's Hospital, Delaware, Wilmington, USA; 3 Allergy and Immunology, Nemours Children's Hospital, Delaware, Wilmington, USA

**Keywords:** asthma, biologics, pediatric asthma management, severe asthma, social determinants of health (sdoh)

## Abstract

Introduction

Pediatric asthma disproportionately impacts under-resourced, minority youth. Monoclonal antibodies (biologics) are a newer treatment option for moderate to severe asthma. Prior work on biologics in pediatric asthma demonstrates improvements in lung function, exacerbations, subjective symptoms, and the need for steroid bursts, but real-world clinical data are limited. The objective of this study was to characterize the demographic profile and clinical outcomes of a cohort of patients with moderate to severe asthma treated with biologic therapy in a single referral center pediatric pulmonology practice.

Methods

This IRB-approved chart review included patients 18 or younger treated with biologic therapy for moderate to severe asthma for at least one year at Nemours Children’s Hospital, a pediatric referral center in Delaware. Zip code was used to determine the area deprivation index (ADI), with higher scores indicating greater disadvantage. Clinical variables at 12 months prior to and after starting treatment were analyzed using a paired two-tailed Student’s t-test, adjusted for multiple comparisons.

Results

Sixteen patients (56% male, 75% Black, 81% non-Hispanic) met the inclusion criteria. Patients started biologics at a median age of 9.5 years. The median ADI was 73.5 (range 13 to 97). Fifty-six percent of patients had public insurance. Median compliance was 96%, ranging from 63% to 100%. There were statistically significant reductions in the number of hospitalizations for asthma (mean pre: 1.63, post 0.25, t = 4.04, p = 0.01) and courses of oral corticosteroids (mean pre: 5.4, post 2.5, t = 4.46, p <0.01). The frequency of ED visits and results on pulmonary function tests improved but did not reach statistical significance.

Conclusions

Patients in our cohort were predominantly non-Hispanic and Black and lived in socioeconomically disadvantaged neighborhoods. Within the first year of treatment with a biologic medication, there was a statistically and clinically significant improvement in hospitalizations, oral corticosteroid courses, and subjective asthma symptoms. Medication adherence and tolerance were excellent.

## Introduction

Asthma is the most common chronic disease in children and has a substantial impact on the quality of life and level of education achieved [1]. Low-income, urban minority youth are disproportionately impacted by asthma [2]. A recent development in the treatment of asthma is the use of monoclonal antibodies, commonly referred to as biologics, to target pro-type 2 (T2) inflammatory pathways in asthma [3]. In children, T2-high disease is the dominant phenotype and is defined by a combination of aeroallergen sensitization, elevated peripheral blood eosinophils, and/or high fractional exhaled nitric oxide (FeNO) levels [4]. The Global INitiative for Asthma (GINA) guidelines recommend biologic therapy for patients who have reached step 5 on the GINA treatment algorithm, which is asthma that is not well controlled on daily medium- to high-dose inhaled corticosteroids (ICS) + long-acting beta 2-agonist, potentially along with other controllers, such as long-acting muscarinic antagonist, leukotriene receptor antagonist, and/or chronic oral corticosteroids [5].

Omalizumab (anti-IgE), dupilumab (anti-IL4 and anti-IL13), benralizumab (anti-IL5 receptor alpha), and mepolizumab (anti-IL5) are approved for children aged six and over, while tezpelumab (anti-thymic stromal lymphopoietin) has been approved for children aged 12 and over. Reslizumab is an anti-IL5 biologic that is only approved for adults >18 years and is given intravenously [6]. Omalizumab is a monoclonal antibody that forms complexes with free serum IgE, reducing the amount of free IgE that is available to bind to mast cells and basophils, thereby decreasing the inflammatory response to allergen exposure [5]. This biologic is indicated in moderate to severe allergic asthma with IgE levels of 30-1300 kU/L in children six to 11 years old and 30-700 kU/L for those 12 and over [4]. Dupilumab was initially approved for atopic dermatitis but is now also indicated for moderate to severe eosinophilic asthma (absolute eosinophil count ≥150-300/μL). Dupilumab blocks the IL-4 receptor alpha, preventing binding of IL-4 and IL-13, which promote the allergic response at multiple stages [4]. Mepolizumab and benralizumab are monoclonal antibodies that bind IL-5 and IL-5 receptor alpha, respectively, and thereby inhibit eosinophilic inflammation [4]. They are indicated for severe asthma with an eosinophilic phenotype, absolute eosinophil count of 150/μL or greater for benralizumab and 300/μL or greater for mepolizumab. Tezpelumab blocks thymic stromal lymphopoietin (TSLP), a cytokine involved in the activation of multiple pro-inflammatory pathways [5]. This medication is indicated as add-on maintenance therapy in severe asthma [4]. There is some evidence that tezpelumab may be useful in non-allergic, non-eosinophilic asthma, given its role in the non-T2 pathway [5]. The decision of which biologic treatment to initiate depends on age, labwork findings, co-morbid conditions, and preferred management strategy based on the frequency of injections, etc. Generally, the GINA guidelines suggest evaluating response to the biologic agent after 4 months with continued re-evaluation every three to six months [6]. If the patient fails to respond to the initial agent but is still eligible for T2 targeted treatment, another biologic agent may be used [6].

Most of the research on biologics in the treatment of asthma has been performed in the adult population [3]. Randomized control trials in the pediatric population have demonstrated improvements in forced expiratory volume, rate of asthma exacerbations, and global ratings of asthma symptoms by physicians and patients [7-9]. Less is known about the real-world clinical outcomes of biologic treatments in pediatric patients with asthma, particularly children under 12 years of age, and how social determinants of health may influence these outcomes. The objective of this study was to characterize the demographic profile and clinical outcomes of a cohort of patients with moderate to severe asthma treated with biologic therapy in a single referral center pediatric pulmonology practice.

## Materials and methods

We conducted a retrospective chart review of information collected as part of standard care. The study was approved by the Institutional Review Board (IRB) at Nemours Children’s Hospital, Delaware, with a waiver of informed consent due to the retrospective nature of the study (approval no. STUDY00000183). The study included all patients aged 18 years and younger who initiated biologic therapy for their moderate to severe asthma and were treated for at least one year. The study included patients who were naïve to biologic treatment as well as those changing to a new biologic treatment. Patients were excluded if therapeutic duration was less than one year at the time of review or if they were treated with biologic therapy predominantly to manage another medical condition (as determined by chart review).

Electronic health records (EHRs) were reviewed manually. Data were extracted and entered into a secure, password-protected Excel spreadsheet in a database that is maintained by the department. Data were manually extracted from EMR medication lists, problem lists (active diagnoses), medication administration records, and clinician notes. Baseline data collected included age, sex assigned at birth, race, ethnicity, zip code, insurance status (private or public), medical conditions, and medications. Compliance was defined as the percentage of treatments received out of the total number of treatments recommended by the physician. Outcome data collected were spirometry data, number of hospitalizations and emergency department visits, and number of steroid courses in the 12 months prior to treatment and 12 months after starting treatment. The zip code was used to determine the area deprivation index. The Area Deprivation Index ranks neighborhoods by socioeconomic disadvantage with consideration of income, education, employment, and housing quality, with higher percentiles indicating more disadvantage [10,11]. Baseline characteristics were summarized with descriptive statistics. Continuous variables were reported as medians with ranges (minimum and maximum). Categorical variables were reported as frequencies and percentages. A paired two-tailed Student’s t-test was used to analyze differences in outcome variables. A Bonferroni correction was applied to account for multiple comparisons.

## Results

Sixteen patients were included in this study (Table 1). Fifty-six percent (9/16) were male, and the median age at starting biologic therapy was 9.5 (ranging from four to 18). Seventy-five percent (12/16) of patients were identified as Black, and 25% (4/16) as White. Eighty-one percent (13/16) identified as non-Hispanic and 19% (3/16) as Hispanic. The median ADI was 73.5, ranging from 13 to 97. 56% (9/16) of the cohort had public insurance. The most common comorbidities were allergic rhinitis (100%, 16/16), atopic dermatitis (56%, 9/16), and obesity (50%, 8/16). Thirty-six percent (6/16) were followed by Psychology during the year of treatment, 31% (5/16) had anxiety, and 25% (4/16) had vocal cord dysfunction.

**Table 1:** Baseline characteristics. Categorical variables are represented as a percentage of the total (n/N). Continuous variables are represented as median (range). Abbreviations: ICS-LABA: inhaled corticosteroid long-acting β2-agonist; LTRA: leukotriene receptor antagonist; n: number of patients in subgroup; N: total number of patients

			% (n/N) or median (range)
Sex	Female		44% (7/16)
	Male		56% (9/16)
Age (years, at start)			9.5 (4-18)
Race	Black		75% (12/16)
	White		25% (4/16)
Ethnicity	Hispanic		19% (3/16)
	Non Hispanic		81% (13/16)
Area Deprivation Index			73.5 (13-97)
Insurance	Public		56% (9/16)
	Private		44% (7/16)
Comorbidities	Allergic rhinitis		100% (16/16)
	Atopic dermatitis		56% (9/16)
	Food allergy		44% (7/16)
	Eosinophilic esophagitis		6% (1/16)
	BMI >95% ile		50% (8/16)
	Obstructive sleep apnea		31% (5/16)
	Anxiety		31% (5/16)
	Vocal cord dysfunction		25% (4/16)
Daily medications	High-dose ICS-LABA		100% (16/16)
	LTRA		69% (11/16)
	Anticholinergic		6% (1/16)
	Every other day steroid		6% (1/16)
Followed by psychology?	Yes, for asthma		0% (0/16)
	Yes, for other reasons		38% (6/16)
	No		62% (10/16)

They started their first year of biologics treatment between 2020 and 2023, and most (15/16) were naive to biologic therapy. Omalizumab was used in seven patients, dupilumab in five, benralizumab in two, mepolizumab in one, and one was started on omalizumab for two months, then switched to dupilumab for an improved side effect profile, concomitant atopic dermatitis, and long-term plans to administer at home. All patients were treated with a high-dose inhaled corticosteroid plus long-acting beta agonist (ICS-LABA). Leukotriene receptor antagonists (montelukast) were prescribed in 69% (11/16) of cases. One patient was on daily anticholinergic therapy (tiotropium bromide), and one was on oral corticosteroids every other day. Median compliance was 96%, ranging from 63% to 100% (Table 2). Reasons for missing doses included delayed authorization or delivery of medication, refusal due to needle phobia, illness, and missed appointments.

**Table 2:** Treatment characteristics Categorical variables are represented as percent of total (n/N). Continuous variables are represented as median (range). Abbreviations: ED: emergency department, EOD: every other day, FEV1: percent predicted forced expiratory volume in one second, FVC: forced vital capacity, ICU: intensive care unit admissions, LTRA: leukotriene receptor antagonist, n: number of patients in subgroup; N: total number of patients

		% (n/N)
Medication	Omalizumab	44% (7/16)
	Dupilumab	37.5% (6/16)
	Benralizumab	12.5% (2/16)
	Mepolizumab	6.3% (1/16)
Compliance	–	96% (63–100%)
Medication changes	None	75% (12/16)
	Added daily anticholinergic	13% (2/16)
	Stopped daily LTRA	6% (1/16)
	Stopped EOD steroid	6% (1/16)
		Median (range)
Oral steroid courses	1 year prior	5 (0–17)
	1 year following	2 (0–11)
ED visits	1 year prior	1 (0–6)
	1 year following	0 (0–3)
Hospitalizations	1 year prior	1 (0–5)
	1 year following	0 (0–4)
ICU admissions	1 year prior	0 (0–2)
	1 year following	0 (0–1)
% pre-FEV₁	1 year prior	83 (64–128)
	1 year following	90 (76–123)
FEV₁/FVC	1 year prior	73 (52–90)
	1 year following	76 (58–92)

There were statistically significant reductions in the number of hospitalizations for asthma per patient (mean pre: 1.63, mean post 0.25, t = 4.04, p = 0.01) and the number of courses of oral steroids (mean pre: 5.4, mean post 2.5, t = 4.46, p < 0.01). Reductions in ED visits and ICU stays did not reach a level of statistical significance, nor did improvements in spirometry data (Table 3).

**Table 3:** Clinical outcomes one year before and one year after biologics treatment for moderate to severe asthma All variables are represented as means. * indicates statistical significance at a level of p < 0.05. P-values from the Student’s t-test were adjusted with Bonferroni method for multiple corrections. Abbreviations: ED: emergency department, FEV1: percent predicted forced expiratory volume in one second, FVC: forced vital capacity, ICU: intensive care unit admissions. Of note, “ED visits” refers to visits that did not lead to hospitalizations only

	Mean pre	Mean post	t-statistic	Adjusted p
Steroid courses	5.38	2.50	4.46	<0.01*
ED visits	1.81	0.81	2.51	0.17
Hospitalizations	1.63	0.25	4.04	0.01*
ICU	0.31	0.06	1.46	1.15
%preFEV1	88.62	92.69	-1.11	2.06
FEV1/FVC	72.13	77.80	-2.14	0.41
% FEV1/FVC ≥ 80	0.23	0.46	-1.79	0.73

One year after starting biologics therapy, one patient was weaned off chronic oral corticosteroids, and one patient stopped montelukast. A daily anticholinergic was added for two of the patients.

## Discussion

In this retrospective chart review, we analyzed the demographic characteristics, clinical features, and clinical outcomes of a cohort of 16 pediatric patients treated with biologic therapy for moderate to severe asthma at a single referral center in Delaware. Our findings highlight the utility of biologic treatment in difficult-to-treat asthma. Within the first year of treatment, there was a statistically significant decrease in the number of hospitalizations and the number of oral corticosteroid courses prescribed compared to the year prior to treatment. Decreases in asthma flares have similarly been reported in large randomized controlled trials of pediatric patients [7-9]. Interestingly, some studies have not found statistically significant changes in spirometry data [9] while others have reported increases in FEV1 [7]. The lack of statistical significance in our study may be attributable to the low sample size of children at the developmental level to participate in spirometry or that the baseline spirometry data for our patients did not show significant impairment, with a mean FEV1 of 88.6% and a mean FEV1/FVC of 72.1%. Consistent with prior work [12], our patients were predominantly non-Hispanic and Black and lived in socioeconomically disadvantaged neighborhoods. In a large cohort of predominantly non-Hispanic and Black individuals in Chicago, greater levels of neighborhood disadvantage have been associated with increased odds of asthma [12]. The American Academy of Asthma, Allergy, and Immunology Committee on the Underserved has several recommendations on improving health equity for individuals with asthma, including implementing community- and school-based health programs, reducing asthma triggers in the physical environment, and improving availability of specialty providers who see Medicaid patients and can prescribe biologic agents [13].

Approximately one in three patients had anxiety. Research suggests that children with asthma have more behavior problems and internalizing disorders (such as anxiety) than their healthy peers, and the association with emotional and behavioral difficulties increases with increasing asthma severity [2]. The 2024 Global Initiative for Asthma (GINA) guidelines recommend assessing for anxiety and depression as comorbidities that may contribute to respiratory symptoms or poor asthma control and referring to psychology or psychiatry as indicated [14].

The limitations of our study include the small sample size and the limitations inherent to chart review, which relies on complete and correct documentation in the EMR. Other studies have shown improved outcomes in patients with longer usage of a biologic agent [8] and with a type 2 inflammatory phenotype [7]. As our patient cohort grows, we anticipate our future work will have sufficient sample size to analyze these characteristics as well.

## Conclusions

In this cohort of children with moderate to severe asthma, there was a statistically significant decrease in the number of hospitalizations and the number of oral corticosteroid courses prescribed within the first year of treatment with a biologic agent, compared to the year prior to treatment. Our patients predominantly lived in socioeconomically under-resourced neighborhoods, and about one-third of patients had a diagnosis of anxiety, underscoring the importance of a multidisciplinary, holistic approach to address environmental and psychosocial determinants of health. The current center now routinely offers referrals to psychology for all patients with severe asthma to assist with co-morbid mental health conditions, medication adherence, educational supports, and the general impact of living with a chronic disease.
